# *Pgc-1α* repression and high-fat diet induce age-related macular degeneration-like phenotypes in mice

**DOI:** 10.1242/dmm.032698

**Published:** 2018-08-16

**Authors:** Meng Zhang, Yi Chu, Joseph Mowery, Brandon Konkel, Susana Galli, Alexander C. Theos, Nady Golestaneh

**Affiliations:** 1 Department of Ophthalmology, Georgetown University Medical Center, Washington, DC 20057, USA; 2 Electron and Confocal Microscopy Unit, USDA Agricultural Research Service, Beltsville, MD 20705, USA; 3 Department of Biochemistry and Molecular and Cellular Biology, Georgetown University Medical Center, Washington, DC 20057, USA; 4 Department of Human Science, Georgetown University, Washington, DC 20057, USA; 5 Department of Neurology, Georgetown University Medical Center, Washington, DC 20057, USA

**Keywords:** RPE, AMD, Retinal degeneration, PGC-1α, High-fat diet, Mitochondria, Autophagy

## Abstract

Age-related macular degeneration (AMD) is the major cause of blindness in the elderly in developed countries and its prevalence is increasing with the aging population. AMD initially affects the retinal pigment epithelium (RPE) and gradually leads to secondary photoreceptor degeneration. Recent studies have associated mitochondrial damage with AMD, and we have observed mitochondrial and autophagic dysfunction and repressed peroxisome proliferator-activated receptor-γ coactivator 1α (PGC-1α; also known as Ppargc1a) in native RPE from AMD donor eyes and their respective induced pluripotent stem cell-derived RPE. To further investigate the effect of PGC-1α repression, we have established a mouse model by feeding *Pgc-1α^+/−^* mice with a high-fat diet (HFD) and investigated RPE and retinal health. We show that when mice expressing lower levels of *Pgc-1α* are exposed to HFD, they present AMD-like abnormalities in RPE and retinal morphology and function. These abnormalities include basal laminar deposits, thickening of Bruch's membrane with drusen marker-containing deposits, RPE and photoreceptor degeneration, decreased mitochondrial activity, increased levels of reactive oxygen species, decreased autophagy dynamics/flux, and increased inflammatory response in the RPE and retina. Our study shows that *Pgc-1α* is important in outer retina biology and that *Pgc-1α^+/−^* mice fed with HFD provide a promising model to study AMD, opening doors for novel treatment strategies.

## Abstract (precis)

**Summary:** A new mouse model has been established that exhibits characteristics of human age-related macular degeneration; the model will facilitate further studies of AMD disease mechanisms.

## INTRODUCTION

Age-related macular degeneration (AMD) is the leading cause of blindness in people over the age of 55 in western countries. Currently, there is no disease-altering treatment for the dry form of AMD, yet millions of patients continue to suffer from this devastating disease. AMD is a multifactorial disease and its etiology results from an interplay between diet, genetics, environmental and metabolic factors (Nowak, 2006).

A number of retinal pathologies including AMD are associated with mitochondrial dysfunction (Karunadharma et al., 2010; Terluk et al., 2015). Dysfunctional mitochondria induce increased levels of reactive oxygen species (ROS) and defective metabolic activity (Jarrett et al., 2013).

Autophagy is a lysosome-mediated degradation process for damaged cellular constituents that supplies the cell with energy and maintains cellular homeostasis under starvation conditions (De Duve, 1963; De Duve and Wattiaux, 1966; Finn and Dice, 2006). Aberrant autophagy also results in mitochondrial dysfunction and accumulation of unwanted organelles, proteins and ROS (Garcia-Prat et al., 2016), eventually leading to cell death (Levine and Yuan, 2005).

Recently, experiments with cells in culture suggested that dysregulated autophagy in retinal pigment epithelium (RPE) increases susceptibility to oxidative stress and the changes associated with AMD (Mitter et al., 2014). In addition, we have described aberrant autophagy, increased ROS and dysfunctional mitochondria in RPE derived from AMD donor eyes (Golestaneh et al., 2017). However, the mechanisms driving these defects in metabolic homeostasis and leading to AMD remain elusive.

The peroxisome proliferator-activated receptor-γ coactivator 1α (PGC-1α; also known as Ppargc1a) has a major role in regulating mitochondrial biogenesis and oxidative metabolism (Liang and Ward, 2006). This protein also regulates autophagy and mitophagy (Vainshtein et al., 2015) and is highly expressed in the healthy retina (Egger et al., 2012). The activity of PGC-1α is stimulated by two metabolic sensors, AMP-activated protein kinase (AMPK) and the NAD^+^-dependent deacetylase SIRT1, through phosphorylation and deacetylation, respectively (Cantó and Auwerx, 2009).

A growing body of evidence suggests that PGC-1α might be responsible for RPE oxidative metabolism and antioxidant defense, retinal light sensibility (Egger et al., 2012; Iacovelli et al., 2016), and the regulation of normal and pathological angiogenesis in the retina (Saint-Geniez et al., 2013); repressed activity of PGC-1α might contribute to AMD (Golestaneh et al., 2016).

To determine whether PGC-1α repression contributes to the pathophysiology of AMD, we focused on age-related changes in the RPE and retina of mice with decreased *Pgc-1α* expression (*Pgc-1α^+/−^* mice).

PGC-1α has an important role in regulating mitochondrial fatty acid oxidation (Vega et al., 2000). In addition, it is reported that a high-fat diet (HFD) can negatively affect retinal function (Chang et al., 2015) and induce diabetic retinopathy (Rajagopal et al., 2016). Hence, we hypothesized that repression of the PGC-1α pathway combined with a HFD would induce degeneration in the RPE and retina, and promote retinal dysfunction.

Here, we have investigated age-related changes in the RPE and retina of *Pgc-1α^+/−^* mice fed with HFD for 4 months. We found that these mice exhibit phenotypes resembling features of human AMD, including lipofuscin accumulation, basal laminar deposits, thickening of Bruch's membrane (BM) with deposits containing oxidative protein damage, and RPE and photoreceptor degeneration. Our data also showed increased expression of drusen-associated genes, decreased mitochondrial DNA (mtDNA) copy number and mitochondrial complex I activity, reduced *Sod2* expression under stress conditions (resulting in increased levels of ROS), decreased autophagy dynamics/flux, and an increased inflammatory response in the RPE/retina of *Pgc-1α^+/−^* mice as compared with wild-type (WT). Hence, our results suggest that repressed levels of PGC-1α, combined with HFD, contribute to the pathophysiology of retinal degeneration and demonstrate a crucial role for PGC-1α in retinal health and function.

## RESULTS

### Increased inflammatory response in *Pgc-1α^+/−^* mice

**Fig. 1.**
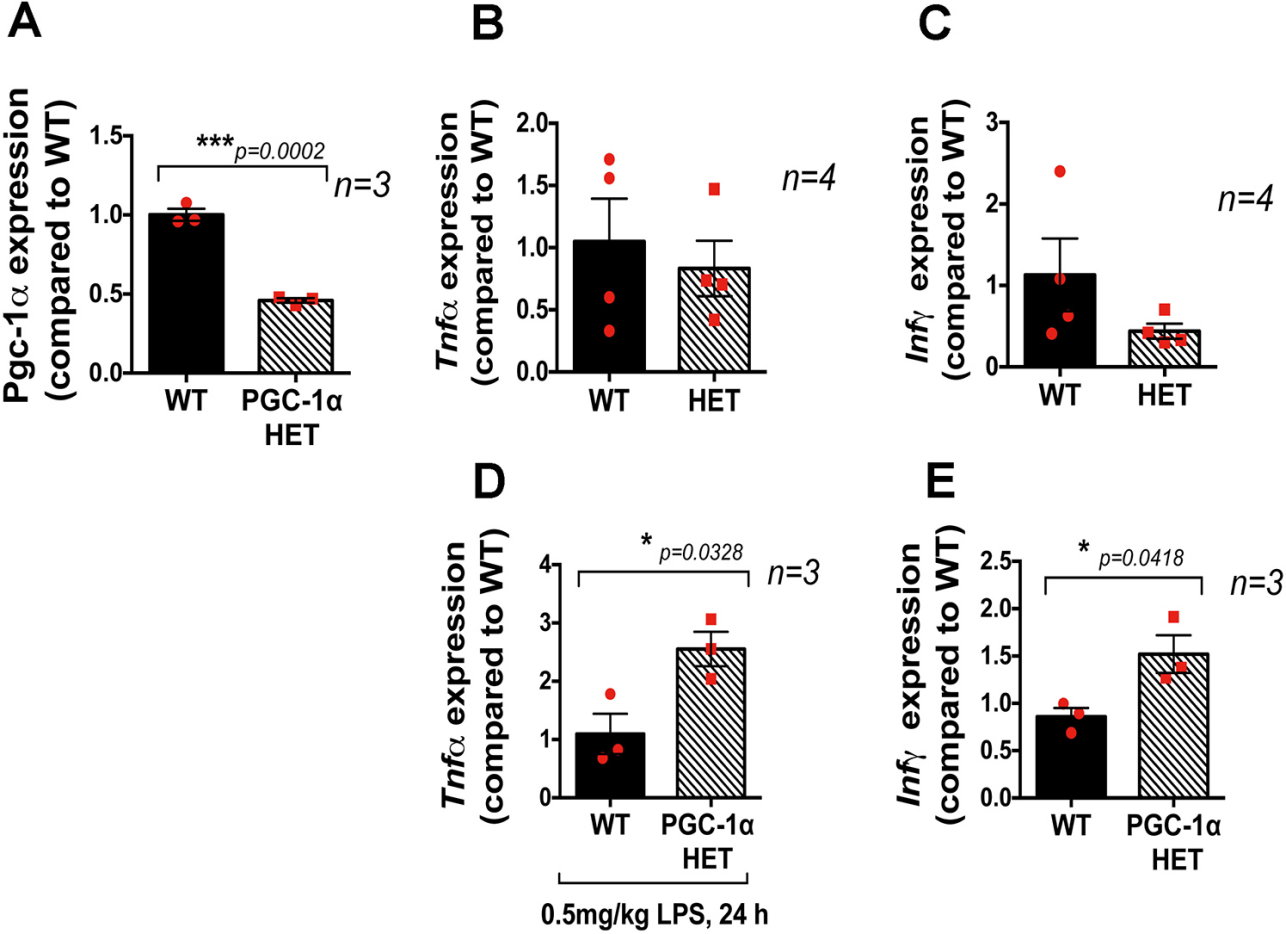
***Pgc-1α^+/−^* mice show an increased inflammatory response to LPS injection.** (A) RPE/retina of *Pgc-1α^+/−^* mice exhibit 50% reduced *Pgc-1α* mRNA expression as compared with WT. (B,C) Basal levels of *Tnfα* and *Infγ* expression in WT and *Pgc-1α^+/−^* (HET) mice in the absence of LPS injection. (D,E) Mice were injected with LPS (0.5 mg/kg, i.p.) and euthanized 24 h after injection. Eyes were enucleated and RPE/retinas extracted for RNA isolation followed by qPCR for *Tnfα* (D) and *Infγ* (E). A higher inflammatory response was observed in the RPE/retina of *Pgc-1α^+/−^* mice compared with WT, as shown by increased expression of *Tnfα* (D) and *Infγ* (E) (*n*=3 WT, *n*=3 *Pgc-1α^+/−^*). One-way ANOVA followed by Student's *t-*test was performed using GraphPad Prism7.

The effect of *Pgc-1α* repression on the inflammatory response was measured by peritoneal injection of lipopolysaccharide (LPS). The retina was extracted 24 h later and quantitative real-time PCR (qPCR) was performed. The inflammatory response was measured by relative expression of the genes encoding tumor necrosis factor α (*Tnfα*; also known as *Tnf*) and interferon γ (*Infγ*; also known as *Ifng*). First, we confirmed that *Pgc-1α* expression was reduced by 50% in the *Pgc-1α^+/−^* mice as compared with WT mice (Fig. 1A). In the absence of LPS there was no difference in the expression levels of *Tnfα* and *Infγ* in the RPE/retina of *Pgc-1α^+/−^* and WT mice (Fig. 1B,C) (*n*=4 WT, *n*=4 *Pgc-1α^+/−^*); however, LPS injection induced a higher inflammatory response in the RPE/retina of *Pgc-1α^+/−^* mice as compared with that of WT (Fig. 1D,E) (*n*=3 WT, *n*=3 *Pgc-1α^+/−^*).

### Accumulation of lipofuscin, basal laminar deposits and loss of fenestrations in the choriocapillaris endothelium in RPE of *Pgc-1α^+/−^* mice fed with HFD

**Fig. 2.**
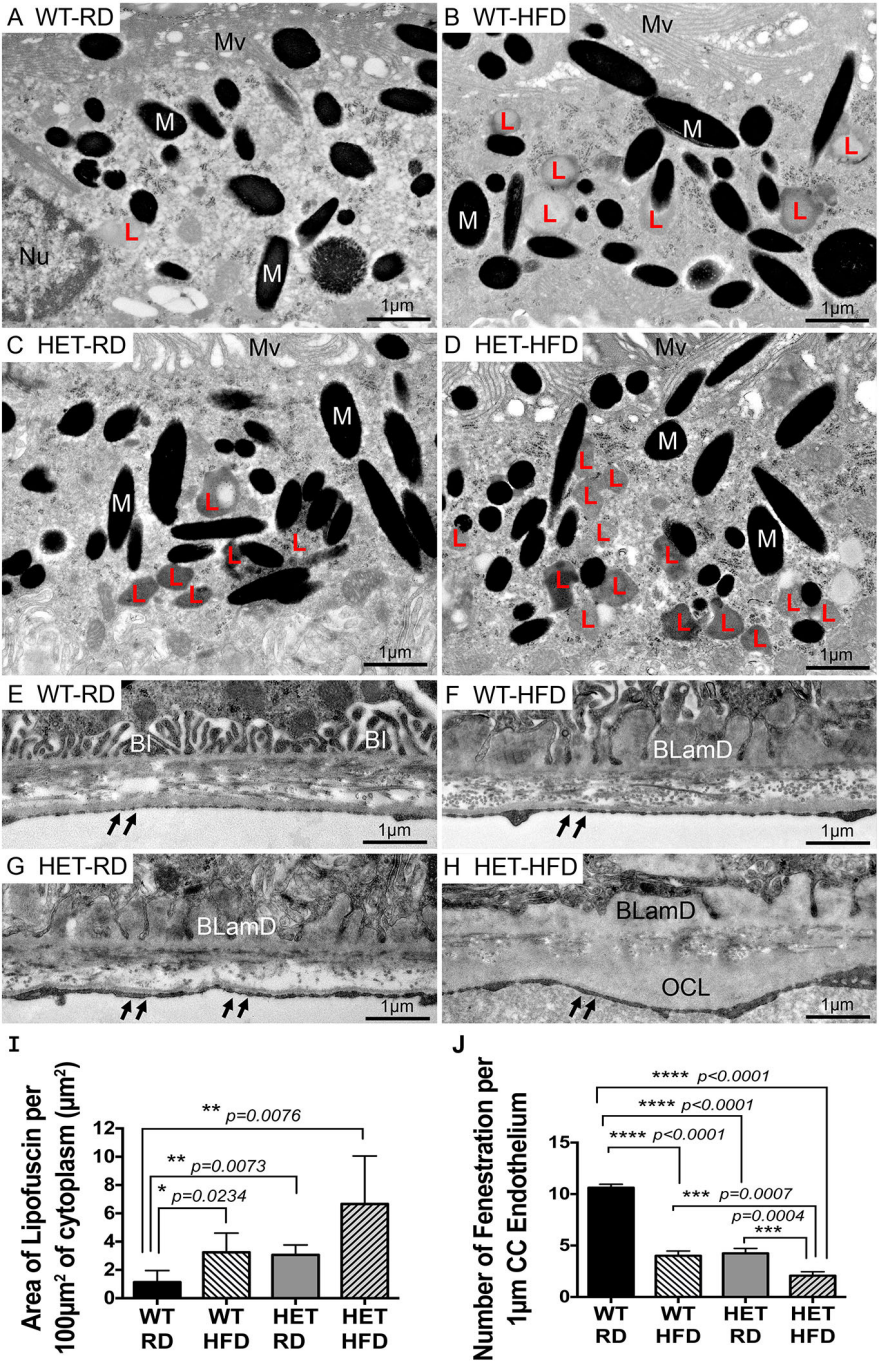
**Accumulation of lipofuscin, basal deposits, thickening of the outer collagenous layer and loss of fenestrations in CC endothelium in RPE of *Pgc-1α^+/−^* mice fed with HFD.** (A-D) Representative TEM images of RPE cytoplasm. (A) WT mice fed RD showing a low amount of lipofuscin. (B) WT mice fed HFD showing elevated amounts of lipofuscin. (C) *Pgc-1α^+/−^* (HET) mice fed RD showing elevated amounts of lipofuscin. (D) HET mice fed HFD showing high amounts of lipofuscin. (E-H) Representative TEM images of BM. (E) WT mice fed RD showing normal basal infoldings and normal fenestrations of the CC endothelium (double arrows). (F) WT mice fed HFD showing an accumulation of basal laminar deposits and fewer fenestrations of the CC endothelium. (G) HET mice fed RD showing an accumulation of basal laminar deposits and fewer fenestrations of the CC endothelium. (H) HET mice fed HFD showing large accumulations of basal laminar deposits, deposits in the OCL layer and loss of fenestrations of the CC endothelium (*n*=5 WT/RD, *n*=5 WT/HFD, *n*=5 HET/RD, *n*=5 HET/HFD). BI, basal infoldings; BLamD, basal laminar deposits; L, lipofuscin; M, melanosomes; Mv, microvilli; Nu, nucleus; OCL, outer collagenous layer. (I) Area of lipofuscin per 100 µm^2^ of cytoplasm, measured from five representative areas from each group and analyzed with Image J analysis software. *Pgc-1α^+/−^* mice fed RD and HFD presented higher number of lipofuscin deposits as compared with WT fed RD. HFD increases the number of lipofuscin deposits in WT. (J) Number of fenestrations per 1 µm of CC endothelium, counted from four representative areas from each group. *Pgc-1α^+/−^* mice fed HFD present the lowest number of CC endothelium fenestrations. One-way ANOVA followed by Dunnett's multiple comparisons test was performed using GraphPad Prism7.

To test whether repression of *Pgc-1α* induces abnormalities in RPE, we investigated the effect of aging under an isocaloric regular diet (RD) and HFD (25% kcal from fat) (Cousins et al., 2002) on RPE and retinal health in *Pgc-1α^+/−^* mice. We fed *Pgc-1α^+/−^* mice at 3 months of age with HFD for 4 months. We used age-matched *Pgc-1α^+/−^* mice and WT mice fed with RD as controls. After 4 months, the animals were euthanized and electron microscopy (EM) performed to analyze RPE morphology. HFD induced abnormalities in both WT (Fig. 2B,F) and *Pgc*-*1α^+/−^* mice (Fig. 2D,H). These abnormalities included the accumulation of lipofuscin in the cytoplasm of RPE and basal laminar deposits (BLamD). However, the severity of these abnormalities appeared to be greater in *Pgc-1α^+/−^* (Fig. 2D,H). In addition, we observed thickening of the outer collagenous layer (OCL), indicative of deposits in *Pgc-1α^+/−^* mice fed HFD (Fig. 2H) as compared with WT mice (Fig. 2F) under the same diet. We also observed loss of fenestration in choriocapillaris (CC) endothelium in *Pgc-1α^+/−^* mice as compared with WT, with increased severity in *Pgc-1α^+/−^* mice fed HFD (arrows in Fig. 2G and H as compared with arrows in Fig. 2E and F). We further quantified the number of lipofuscin deposits per 100 µm^2^ of cytoplasm and the number of fenestrations per 1 µm of CC endothelium. We observed increased numbers of lipofuscin deposits caused by HFD in WT (Fig. 2B,I). *Pgc-1α^+/−^* mice fed RD showed higher numbers of lipofuscin deposits, as compared with WT fed RD, that were similar to the levels observed in WT mice fed HFD (Fig. 2I). Therefore, HFD and *Pgc-1α* repression can both cause lipofuscin formation and if combined they could have an additive effect. Within our samples, however, the trend in increased lipofuscin was not statistically significant between WT and *Pgc-1α^+/−^* mice fed HFD or between *Pgc-1α^+/−^* mice fed RD and HFD. The number of fenestrations per 1 µm of CC endothelium was significantly reduced in WT mice fed HFD, as compared with WT fed RD, indicating that HFD can negatively affect CC endothelium (Fig. 2J). In addition, repression of *Pgc-1α* combined with HFD further reduced the number of fenestrations in *Pgc-1α^+/−^* mice fed HFD, as compared with *Pgc-1α^+/−^* mice fed RD (Fig. 2J).

### Repression of *Pgc-1α* combined with HFD induces damage to RPE, photoreceptors and BM, and promotes enlarged choroidal blood vessels

**Fig. 3.**
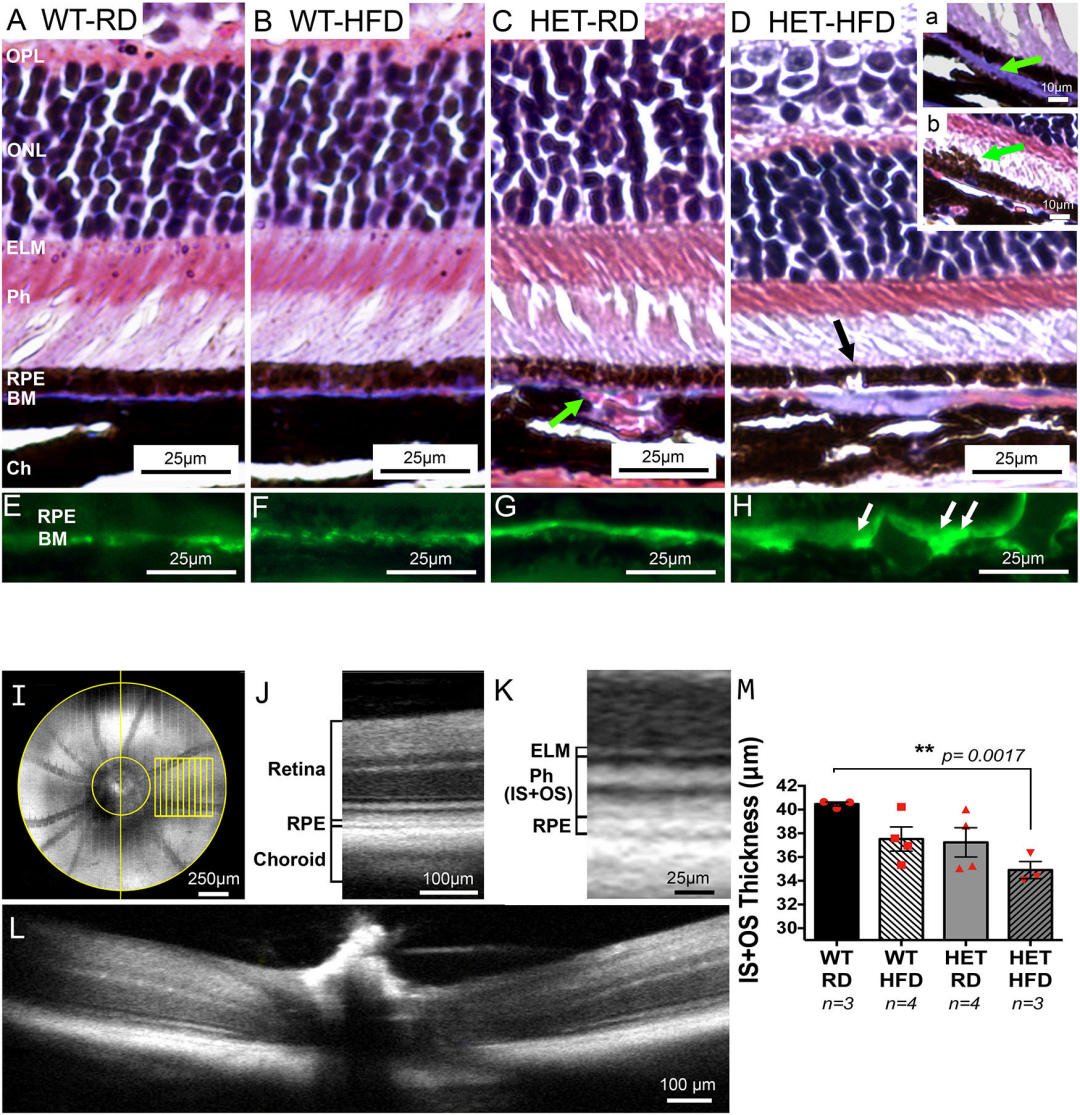
**Repression of *Pgc-1α* combined with HFD induces damage to RPE, photoreceptors and BM; enlarged choroidal blood vessels are also observed.** Eyes from WT and *Pgc-1α^+/−^* mice fed RD and HFD were fixed, paraffin-embedded and stained with Masson's Trichrome. No changes were observed in the RPE or retinal structures in WT fed (A) RD or (B) HFD. (C) *Pgc-1α^+/−^* (HET) mice fed RD showed enlarged and congested blood vessels in the interface between the BM and the choroid (green arrow). (D) *Pgc-1α^+/−^* mice fed with HFD exhibit enlarged choroidal blood vessels. Changes in the BM, characterized either by atrophy or thickening of the membrane (inset a; green arrow). RPE degeneration was observed with breaks or ‘gaps’ (black arrow) and occasional melanosomes migrating into the outer segment (inset b; green arrow). Photoreceptor degeneration was apparent in the *Pgc-1α^+/−^* mice fed HFD (D) presented by reduced thickness of the photoreceptor layer as compared with WT fed RD (A) or HFD (B) and *Pgc-1α^+/−^* mice fed with RD (C). (E-H) Immunofluorescence staining with CML, a drusen marker, in WT fed RD (E), WT fed HFD (F), *Pgc-1α^+/−^* fed RD (G) and *Pgc-1α^+/−^* fed HFD (H), showing accumulation of CML deposits in the BM of *Pgc-1α^+/−^* mice fed HFD (H; white arrows). (*n*=5 WT/RD, *n*=5 WT/HFD, *n*=5 HET/RD, *n*=5 HET/HFD). (I) *En face* image of a mouse eye obtained by OCT with the 0.5 mm×0.5 mm approximate sampling locations (in yellow) relative to the optic nerve. (J) Representative OCT B-scan with the defined retina and adjacent layers identified. (K) Magnified region of the representative B-scan with the defined IS+OS layers and bounding layers identified. ELM, external limiting membrane; Ph (IS+OS), inner and outer segments of the photoreceptor layer. (L) B-scan corresponding to the vertical yellow line from the *en face* image (I), demonstrating the 0.5 mm diameter (area between yellow lines) around the optic nerve excluded from sampling to remove bias from layer curvature in this area. (M) Measurement of IS+OS thickness in µm. *Pgc-1α* repression combined with HFD induces photoreceptor degeneration. One-way ANOVA followed by Dunnett's multiple comparisons test was performed using GraphPad Prism7.

We performed three-color Masson's Trichrome staining to identify damage in RPE, photoreceptors, BM and choroid. We identified enlarged blood vessels in the interface between the BM and the choroid, with congestion and dilatation of some vessels (arrow Fig. 3C) in the *Pgc-1α^+/−^* mice as compared with WT (Fig. 3A,B). We observed changes in the BM, characterized as either atrophy or thickening in various regions (Fig. 3D, green arrow inset a), in the *Pgc-1α^+/−^* mice fed with HFD as compared with WT under the same diet. In addition, we observed RPE degeneration with disruptions or ‘gaps’ and scant melanosomes in the subretinal space migrating into the outer segment in *Pgc-1α^+/−^* mice fed with HFD (black arrow Fig. 3D, green arrow inset b) as compared with WT under the same diet. Degeneration of the photoreceptor layer was noticeable, as the layer was thinner in *Pgc-1α*^+/−^ mice fed with HFD when compared with *Pgc-1α*^+/−^ mice fed with RD or WT mice fed with either RD or HFD (Fig. 3D). No inflammatory infiltrate was observed. To further demonstrate drusen deposits underneath the RPE, we performed immunofluorescence staining with the anti-carboxymethyl lysine (anti-CML) antibody (Fig. 3E-H). CML is a marker of oxidative protein damage and is found in macular drusen in AMD (Ishibashi et al., 1998). We observed accumulation of CML deposits in the thickened BM of the *Pgc-1α^+/−^* mice fed HFD, suggesting that *Pgc-1α* repression combined with HFD induces oxidative damage in BM (Fig. 3H). Basal laminar deposits could also trap drusen components during passage from RPE to BM. The CML staining further supports our EM observations that show basal laminar deposits in *Pgc-1α^+/−^* mice fed with HFD.

To quantify photoreceptor degeneration, we performed optical coherence tomography (OCT). Fig. 3I-L describe how we acquired the images for measurement of the photoreceptor inner segment (IS) and outer segment (OS). Analyses of the right eyes showed a significant reduction of thickness of the IS+OS layer in *Pgc-1α^+/−^* mice fed with HFD as compared with WT fed RD (Fig. 3M; *P*=0.0017). Analyses of the left eyes showed a trend towards reduced IS+OS thickness; however, the differences were not statistically significant in our samples. This observation suggests that occurrence of the AMD-like phenotype in mice does not necessarily initiate concurrently in both eyes, which more accurately reflects the condition in humans. We also observed reduced IS+OS thickness in WT mice fed HFD and *Pgc-1α^+/−^* mice fed RD, as compared with WT control, suggesting that either HFD or repressed *Pgc-1α^+/−^* alone might induce photoreceptor degeneration to some extent; however, the differences were not statistically significant within our samples (*P*=0.05 and *P*=0.07, respectively).

### Repression of *Pgc-1α* induces expression of drusen-associated genes in the RPE/retina

**Fig. 4.**
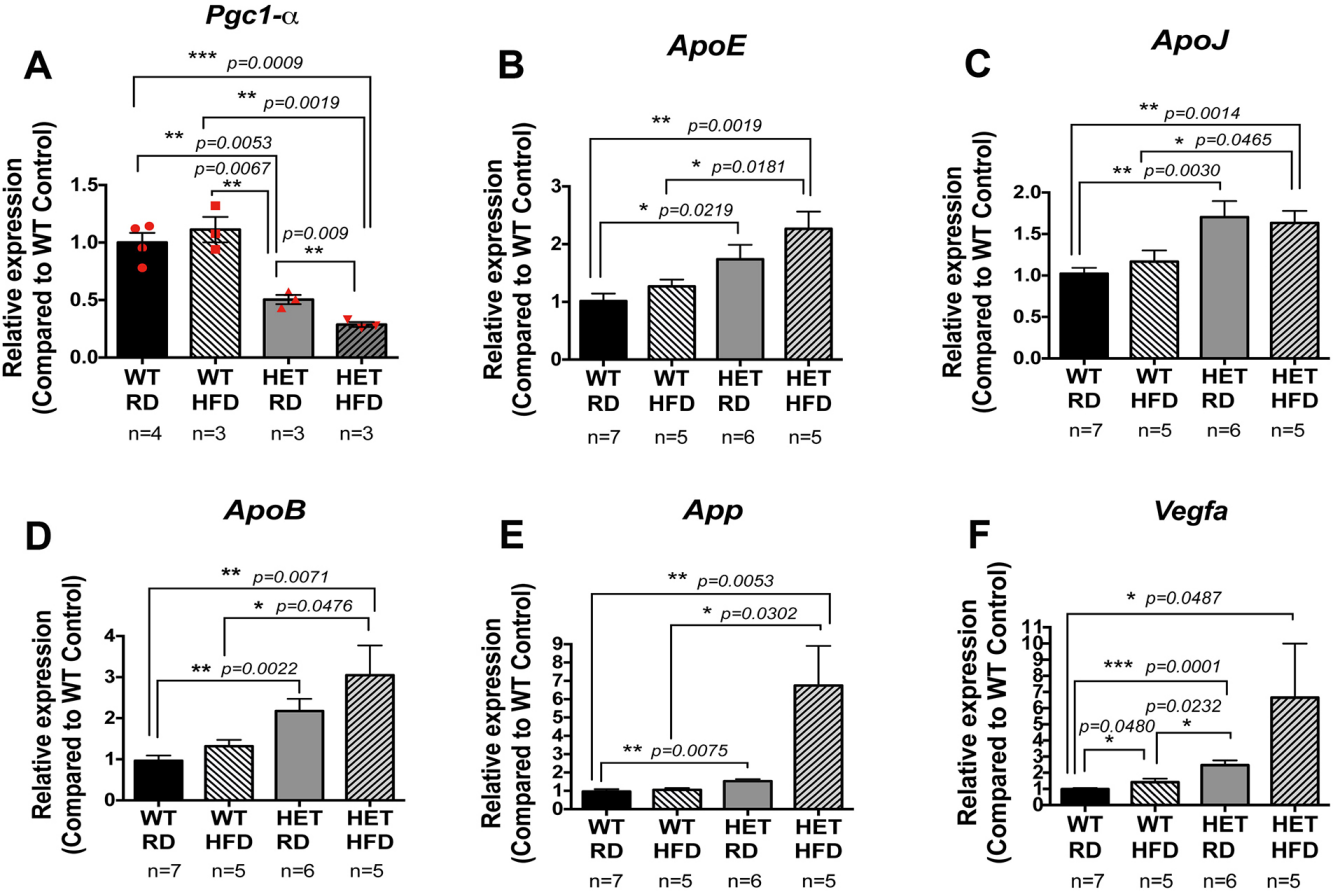
**Increased expression of the drusen-associated genes (*ApoE*, *ApoJ*, *ApoB* and *App*) and *Vegfa* in the RPE/retina of *Pgc-1α^+/−^* mice under RD and HFD.** (A) The expression of *Pgc-1α* is reduced in the RPE/retina of *Pgc-1α^+/−^* (HET) mice fed HFD as compared with *Pgc-1α^+/−^* mice fed RD. (A-E) The expression of drusen-associated genes *ApoE* (B), *ApoJ* (C), *ApoB* (D) and *App* (E) is increased in the RPE/retina of *Pgc-1α^+/−^* fed RD or HFD, as compared with WT mice. (F) *Pgc-1α* repression induces *Vegfa* expression, as shown by increased levels of *Vegfa* in RPE/retina of *Pgc-1α^+/−^* mice fed RD as compared with WT. HFD seemed to increase *Vegfa* expression in WT, but levels were still lower than those in *Pgc-1α^+/−^*. One-way ANOVA followed by Dunnett's multiple comparisons test was performed using GraphPad Prism7.

Drusen are hallmarks of dry AMD. Because PGC-1α has a central role in lipid metabolism and fatty acid β-oxidation (Li et al., 2008; Nikolic et al., 2012), and as we observed accumulation of CML deposits in the BM of *Pgc-1α^+/−^* mice, we tested the effect of *Pgc-1α* repression on the expression of drusen-associated genes in the RPE/retina of mice fed with RD or HFD. We first tested the effect of HFD on *Pgc-1α* expression in the RPE/retina of WT and *Pgc-1α^+/−^* mice. Our data showed that in *Pgc-1α^+/−^* mice, where *Pgc-1α* expression is 50% less than WT, the HFD further reduced *Pgc-1α* expression in the RPE/retina as compared with WT (Fig. 4A). This finding suggests that HFD could have negative regulatory effects on *Pgc-1α* expression when the expression is already reduced. We then used qPCR to analyze expression of the genes encoding three apolipoproteins [*ApoE*, *ApoJ* (also known as Clu) and *ApoB*] (Li et al., 2006) and the amyloid precursor protein (*App*), proteolysis of which generates β amyloid (Aβ) a component of senile plaque and drusen (Luibl et al., 2006). APOB is the major apolipoprotein in low-density lipoprotein cholesterol and has been identified in cholesterol-containing drusen and basal deposits of human eyes with age-related maculopathy (Malek et al., 2003). Mice expressing the human form of APOB100 have been proposed as an animal model for dry AMD (Pennesi et al., 2012).

We observed an increase in the expression of the drusen-associated genes including *ApoE*, *ApoJ*, *ApoB* and *App* in the RPE/retina of *Pgc-1α^+/−^* mice as compared with WT, suggesting that *Pgc-1α* negatively regulates the expression of these genes (Fig. 4B-E)*.* HFD induced an increase in expression of drusen-associated genes in WT, although this was not statistically significant within our samples. Nevertheless, the expression of drusen-associated genes kept its higher levels in *Pgc-1α^+/−^* mice fed HFD as compared with WT under the same diet, suggesting that HFD alone might increase the expression of drusen-associated genes to a certain extent.

Because we observed enlarged blood vessels in the choroid of *Pgc-1α^+/−^* mice, we tested the expression of vascular endothelial growth factor A (*Vegfa*), which is responsible for neovascularization in AMD. Our data showed that expression of *Vegfa* is significantly higher in the RPE/retina of *Pgc-1α^+/−^* mice fed RD or HFD as compared with WT under the same diet, indicating that *Pgc-1α* can negatively regulate *Vegfa* (Fig. 4F). In addition, HFD was able to increase the expression of *Vegfa* in WT, indicating that HFD alone can induce *Vegfa* expression in RPE/retina.

### Increased ROS levels, an inability to induce antioxidant response, and damaged and dysfunctional mitochondria in the RPE/retina *of the Pgc-1α^+/−^* mice fed HFD

PGC-1α has a crucial role in maintenance of the mitochondrial antioxidant defense system (Valle et al., 2005), mitochondrial biogenesis and turnover (St-Pierre et al., 2006, 2003), and controls detoxification of ROS by upregulating the expression of ROS-detoxifying enzymes (Austin et al., 2011; St-Pierre et al., 2003).

**Fig. 5.**
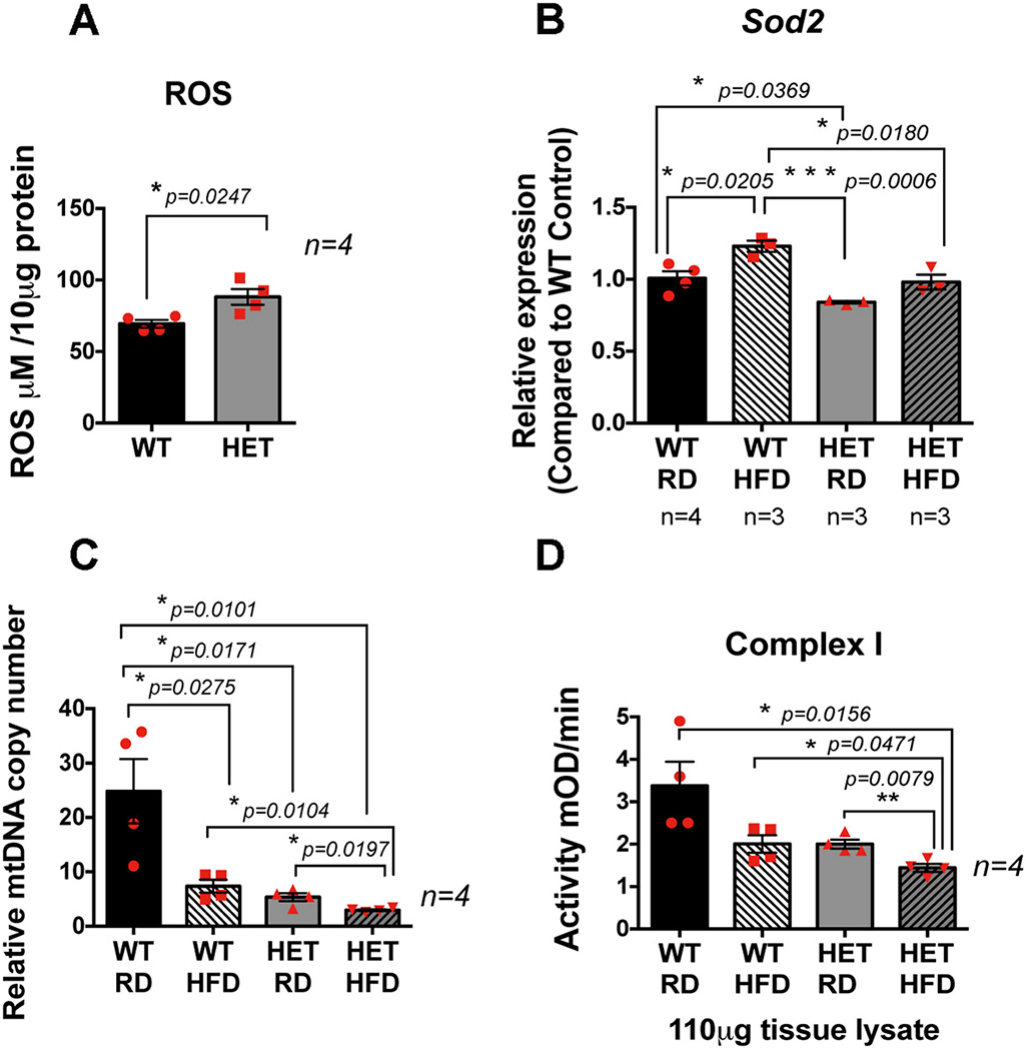
**Reduced antioxidant capacity in RPE/retina of *Pgc-1α^+/−^* mice, and decreased mitochondrial activity in RPE/retina of *Pgc-1α^+/−^* mice fed HFD.** (A) ROS measurement in the RPE/retina extract of WT and *Pgc-1α^+/−^* mice showing increased ROS levels in the *Pgc-1α^+/−^* mice as compared with WT. (B) Reduced antioxidant capacity in the RPE/retina of *Pgc-1α^+/−^* mice, as compared with WT, shown by reduced *Sod2* expression and inability to induce *Sod2* expression under stress conditions such as HFD. (C) mtDNA copy number measured by qPCR showing reduced mtDNA induced by HFD. *Pgc-1α* repression combined with HFD further reduced the mtDNA copy number. (D) *Pgc-1α* repression and HFD significantly reduced mitochondrial complex I activity.

We measured ROS levels in the RPE/retina of WT and *Pgc-1α^+/−^* mice. Our data showed increased levels of ROS in the RPE/retina of *Pgc-1α^+/−^* mice as compared with WT (Fig. 5A). We further analyzed *Sod2* expression levels in the RPE/retina of WT and *Pgc-1α^+/−^* mice fed RD or HFD. Our data showed that *Sod2* levels under normal conditions (RD) were significantly lower in the RPE/retina of *Pgc-1α^+/−^*, as compared with WT mice (Fig. 5B). In addition, although WT mice could significantly increase *Sod2* levels under stress conditions, identified as the HFD, the *Pgc-1α^+/−^* mice were unable to do so (Fig. 5B).

To investigate the effect of *Pgc-1α* repression and HFD on mitochondrial damage and function we analyzed the mtDNA copy number in the RPE/retina of *Pgc-1α^+/−^* mice fed RD or HFD, as compared with WT mice. Our data revealed that HFD significantly reduced mtDNA copy number independent of genotype in the RPE/retina of WT and *Pgc-1α^+/−^* mice, and repression of *Pgc-1α* further decreased the mtDNA copy number (Fig. 5C).

To further analyze mitochondrial function, we measured mitochondrial complex I activity in the RPE/retina of WT and *Pgc-1α^+/−^* mice fed RD or HFD. Our data showed a significant decrease in complex I activity in the *Pgc-1α^+/−^* mice fed HFD as compared with WT fed RD, WT fed HFD and *Pgc-1α^+/−^* fed RD (Fig. 5D).

### Autophagy dynamics and flux are reduced in the retina of *Pgc-1α^+/−^* mice

Dysfunctional autophagy is likely to be associated with AMD (Mitter et al., 2014) and we have previously shown that autophagy is dysfunctional in RPE derived from AMD donors (Golestaneh et al., 2017). Because PGC-1α is reported to regulate autophagy (Halling et al., 2016; Vainshtein et al., 2015), we quantitated autophagy dynamics in the RPE/retina of *Pgc-1α^+/−^* and WT mice by monitoring the ubiquitin-like microtubule-associated protein 1 light chain LC3-I, which after lipidation becomes LC3-II (a commonly accepted method to measure autophagy; Mizushima and Yoshimori, 2007).

**Fig. 6.**
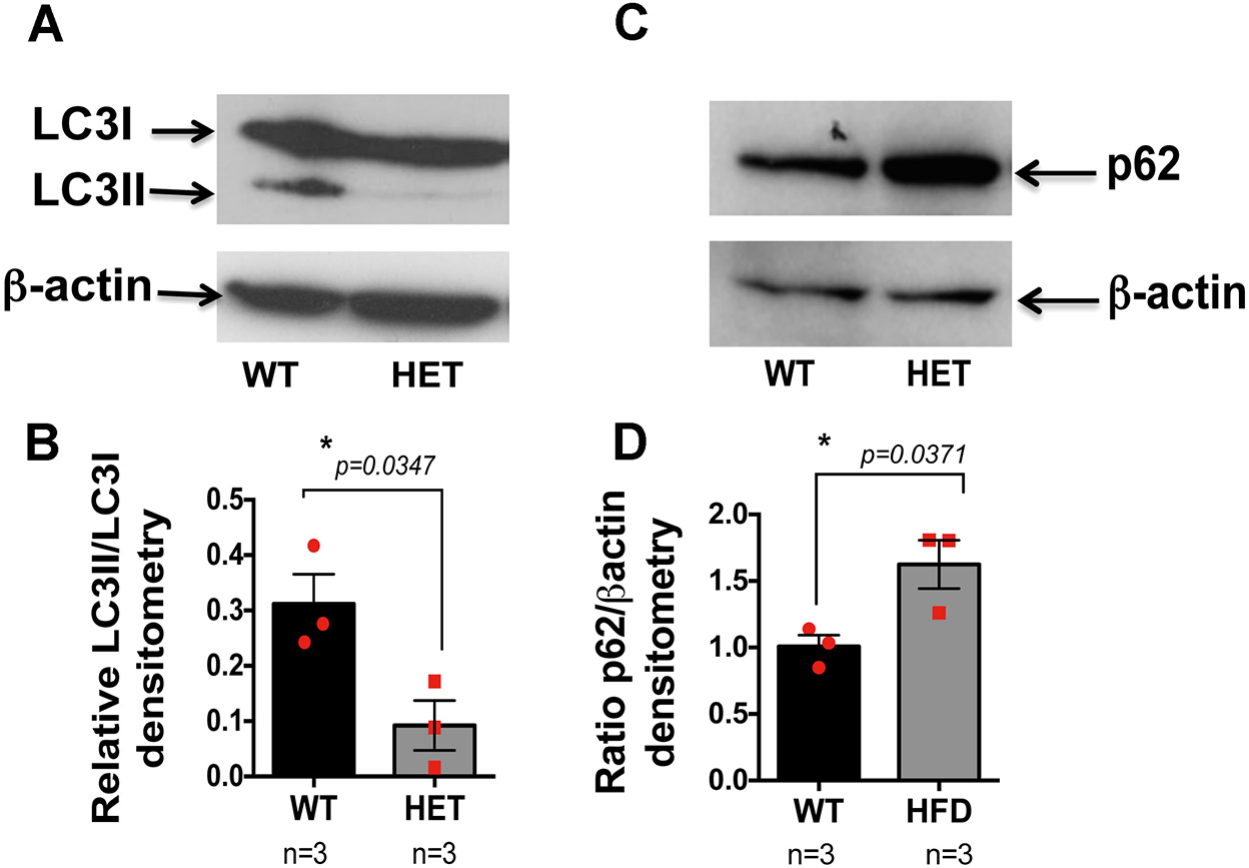
**Autophagy dynamics and flux are reduced in the RPE/retina of the *PGC-1α^+/−^* mice.** (A,C) Western blot analysis with anti-LC3 (A) and p62 (C) antibodies (compared with β-actin loading control) revealed reduced autophagy dynamics (A) and flux (C) in the RPE/retinal protein extract of *Pgc-1α^+/−^* (HET) mice, as compared with WT (a representative image of three independent experiments). (B,D) Densitometry analysis of A and C, respectively, (*n*=3 WT; *n*=3 HET) performed using ImageJ. One-way ANOVA followed by Dunnett's multiple comparisons test was performed using GraphPad Prism7.

Our data showed that autophagy is reduced in the RPE/retina of *Pgc-1α^+/−^* mice compared with WT, as shown by the ratio of LC3II/LC3I (Fig. 6A,B). We also tested the autophagy flux by analyzing the p62, also called sequestosome 1 (SQSTM1), that is degraded by autophagy and is considered a marker of autophagic flux (Bjørkøy et al., 2005). Our data demonstrated that levels of p62 are increased in the RPE/retina of *Pgc-1α^+/−^* mice as compared with WT (Fig. 6C,D). These findings are consistent with the abnormalities observed in the RPE of *Pgc-1α^+/−^* mice by EM, and correlate with our findings in human RPE with AMD (Golestaneh et al., 2017).

## DISCUSSION

Recently, it was reported that PGC-1α induces human RPE oxidative metabolism and antioxidant capacity and rescues human RPE from oxidative damage (Iacovelli et al., 2016). PGC-1α also regulates normal and pathological angiogenesis in the retina and determines light-damage susceptibility in *Pgc-1α^−/−^* mouse retina (Egger et al., 2012; Saint-Geniez et al., 2013). Variants in PGC-1α are associated with neovascular AMD and AMD-associated loci (SanGiovanni et al., 2013). However, the complete absence of the gene product does not reflect conditions in humans. Here, we investigated the role of *Pgc-1α* repression combined with dietary influence on retinal health and AMD-associated phenotypes.

Our study indicates that the RPE and retina of *Pgc-1α^+/−^* mice fed with HFD exhibit features of the RPE and retina of AMD donor eyes and recapitulate many characteristics of AMD pathophysiology. We demonstrate that the retinas of *Pgc-1α^+/−^* mice show increased inflammatory responses to peritoneal LPS injection. As *Pgc*-1α has an important role in lipid metabolism and fatty acid β-oxidation (Li et al., 2008; Nikolic et al., 2012), we fed the mice with HFD to investigate the effect of diet and aging on RPE and photoreceptor health. We observed that *Pgc-1α^+/−^* mice fed with HFD exhibited increased numbers of lipofuscin deposits in the RPE, basal laminar deposits, BM thickening and outer collagenous layer deposits; these mice also showed a reduced number of fenestrations in the CC endothelium. In addition, our data showed RPE and photoreceptor degeneration, and scant melanosomes migrating into the outer segment in the *Pgc-1α^+/−^* mice.

Accumulation of lipofuscin has been associated with aging and AMD (Ach et al., 2015; Suter et al., 2000) and its pharmacological inhibition has been proposed as a therapeutic strategy in dry AMD treatment (Petrukhin, 2013). However, a new study showed redistribution and loss of autofluorescent granules in the RPE of eyes with AMD (Ach et al., 2015); therefore, the role of lipofuscin in AMD remains controversial and further research is needed to delineate the mechanism by which the accumulation or loss of autofluorescent granules could contribute to AMD.

Thickening or atrophy of BM with loss of interaction within the photoreceptor-RPE-BM-CC complex is one of the features of AMD (Bhutto and Lutty, 2012; Chong et al., 2005; Majji et al., 2000) that results in degeneration of all of the layers in the complex (Bhutto and Lutty, 2012).

Drusen are hallmarks of AMD, and the expression of drusen-associated genes is increased in RPE under conditions of oxidative stress and in AMD (Golestaneh et al., 2017; Rabin et al., 2013). In our study, we observed increased expression of drusen-associated genes in the RPE/retina of *Pgc-1α^+/−^* mice fed with RD and HFD, as compared with WT under the same diets. Accordingly, we observed increased laminar deposits and accumulation of CML deposits in the thickened BM in the *Pgc-1α^+/−^* mice fed HFD, suggesting that *Pgc-1α* repression combined with HFD induces oxidative protein damage in mice. We euthanized and analyzed the *Pgc-1α^+/−^* mice fed HFD at 7 months of age and did not observe dome-shaped drusen structures. In another study in *Sod1^−/−^* mice, the authors did not find dome-shaped drusen in mice at 7 months of age and could only see drusen in mice older than 7 months. By contrast, WT and heterozygote (*Sod1^+/−^*) mice did not show significant drusen formation (Imamura et al., 2006). Light exposure in a box surrounded by mirrors for 24 h per day for 8 weeks with 10,000 lux light intensity induced dome-shaped drusen formation in younger *Sod1^−/−^* mice (Imamura et al., 2006).

Here, we studied the heterozygote (*Pgc-1α^+/−^*) mice fed with HFD and have observed basal laminar deposits and thickened BM with deposits containing the drusen marker CML. It is possible that these mice at an older age or under certain stress conditions could also present dome-shaped drusen formation.

Choroidal neovascularization occurs in the wet form of AMD. An individual could present both forms, however, or initially develop dry AMD that could later transform into wet AMD. We have also noticed enlarged choroidal blood vessels and reduced numbers of fenestrations in the CC endothelium in *Pgc-1α^+/−^* mice fed RD and HFD with more severe loss of CC fenestration in *Pgc-1α^+/−^* mice fed HFD, as compared with WT mice under the same conditions. Consistent with the microscopic analysis we also observed increased *Vegfa* gene expression in the RPE/retina of *Pgc-1α^+/−^* mice as compared with WT. In addition, we observed that HFD alone could induce *Vegfa* expression independently of genotype.

Mitochondrial damage and lower mtDNA copy number have been associated with AMD (Golestaneh et al., 2016, 2017; Karunadharma et al., 2010; Nashine et al., 2017; Terluk et al., 2015). We tested mtDNA copy number and mitochondrial complex I enzyme activity and observed the lowest mtDNA copy number and complex I activity in the *Pgc-1α^+/−^* mice fed HFD, as compared with WT fed RD. Whereas HFD seemed to have a negative effect on mtDNA, the combination of *Pgc-1α* repression and HFD appeared to exert an accumulative effect on both mtDNA and complex I activity.

We also tested the effect of *Pgc-1α* repression on RPE function by analyzing autophagy in the RPE/retina. As PGC-1α regulates autophagy and mitophagy, we can postulate that repression of PGC-1α activity could result in AMD-associated phenotypes through inhibition of autophagy in RPE and retina. Our data strongly support this hypothesis and show that the autophagy pathway is inhibited in the RPE/retina of *Pgc-1α^+/−^* mice. Moreover, we observed accumulation of lipofuscin and dysfunctional mitochondria in the RPE/retina of *Pgc-1α^+/−^* mice fed with HFD, consistent with dysfunctional autophagy. These features have also been identified in the RPE of AMD donors in our previous study (Golestaneh et al., 2017). The increased expression of drusen-associate genes and *Vegfa* in the RPE/retina of *Pgc-1α^+/−^* mice under RD and HFD also suggests that *Pgc-1α* might regulate the expression of these genes. Thus, repression of PGC-1α could affect drusen formation through its regulatory role on autophagy, which would result in the accumulation of unwanted materials, and by influencing the expression of genes implicated in drusen formation.

Recent studies have reported on CC endothelium breakdown preceding retinal degeneration and loss of CC endothelium in early AMD (Biesemeier et al., 2014; Whitmore et al., 2015). Several AMD animal models have been created through the deletion or overexpression of a specific gene or using a combination of environmental factors such as smoking, HFD and blue light to model characteristics of AMD (Pennesi et al., 2012), each addressing a specific aspect of the disease. However, no animal model fully recapitulates AMD features, which are characterized by loss of the photoreceptor-RPE-BM-CC complex (Bhutto and Lutty, 2012). Here, we have generated a mouse model, *Pgc-1α^+/−^* mice fed HFD, that recapitulates multiple features of human AMD. Importantly, this model exhibits the breakdown of the CC endothelium through loss of fenestrations, laminar deposits, outer collagenous layer deposits causing BM thickening (with the deposits containing oxidative protein damage), and RPE and photoreceptor degeneration; this model could be proposed as a dry AMD model. However, because of increased *Vegfa* expression and enlarged blood choroidal vessels, this model could also be useful for studies of early stage wet AMD.

In conclusion, our study demonstrates that repression of the *Pgc-1α* gene, in combination with aging and environmental factors such as diet, can induce AMD-like phenotypes in mice. These animals exhibit accumulation of lipofuscin, basal and outer collagenous layer deposits, BM thickening and CML-containing deposits, enlarged blood vessels and loss of CC endothelium fenestrations. In addition, these mice show signs of RPE and photoreceptor degeneration, express increased levels of drusen-associated genes and *Vegfa*, exhibit reduced antioxidant defense and mitochondrial capacity and function, and demonstrate decreased autophagy dynamics in the RPE/retina. Our study opens new doors to validate novel therapies for AMD and proposes a new animal model to study the disease.

## MATERIALS AND METHODS

### Mice

*Pgc-1α^+/−^* mice on a C57BL/6 background were generated by crossing *Pgc-1α^+/−^* and WT C57BL/6 mice purchased from the Jackson Laboratory. Genotyping was performed by DNA extraction from mouse-tail tissue using Wizard SV Genomic DNA purification system (Promega), followed by PCR (Applied Bio-systems). The results were analyzed using agarose gel electrophoresis and viewed under UV light with MyECL Imager (Thermo Fisher Scientific). All animals were used in compliance with the Statement for the Use of Animals in Ophthalmology and Visual Research from the Association for Research in Vision and Ophthalmology (ARVO), and performed under an approved protocol by the Georgetown University Institutional Animal Care and Use Committee (GU.IACUC, protocol 2016-1146).

### High-fat diet

*Pgc-1α^+/−^* mice and WT mice both male and female at 3 months of age were fed with HFD for 4 months. HFD was formulated to provide 25% kcal from fat (TestDiet). The RD was an isocaloric diet (TestDiet) providing 11% kcal from fat. After 4 months we performed OCT and euthanized the animals for biochemical and molecular analyses.

### Induction of inflammation

WT (*n*=3) and *Pgc-1α^+/−^* (*n*=3) mice were injected intraperitoneally with LPS at 0.5 mg/kg and euthanized 24 h later. Eyes were enucleated and the retina and RPE were isolated and prepared for RNA extraction according to the manufacturer's protocol (Thermo Fisher Scientific).

### Optical coherence tomography

Mice were anesthetized with intraperitoneal injection of a cocktail of xylazine (5-10 mg/kg), acepromazine (2-3 mg/kg) and ketamine (65-100 mg/kg) approximately 10 min before imaging. Pupils were dilated with 1 drop per eye of 1% tropicamide (Alcon; pharmaceutical grade, sterile solution) and 2.5% phenylephrine hydrochloride (Ciba Vision; pharmaceutical grade, sterile solution) 3-5 min before imaging. Mice were mounted and secured to a stereotaxic device to stabilize and orient the head. Mice were prepared for imaging using the protocol described by Sen et al. (2016). A plastic O-ring was secured with a fluid-tight seal around the eye using vacuum grease (Dow Corning), filled with 2.5% hypromellose solution (Gonak™) and covered by a 0.17 mm thick coverslip.

Images were captured using a Ganymede™ High-Resolution SD-OCT system (ThorLabs) with a 930 nm center wavelength equipped with an LSM03 objective, providing an axial resolution of 8 μm. Image sets were captured in 3D with mice oriented to center the optic nerve in the captured volume. Images were captured at an A-scan rate of 36 kHz with A-scan averaging of 4. Image volumes were approximately 2.5 mm×2.5 mm with a depth of 1.39 mm (after adjusting for a refractive index of 1.4).

Image sets were anonymized and images segmented in ImageJ by two blind manual raters. Each rater was presented with 10 B-scans spaced 50 μm apart, spanning a 0.5 mm region adjacent to the optic nerve (Fig. 3I). Raters segmented the top of the retina, the top of the IS+OS of the photoreceptor layer where the IS intersects with the external limiting membrane (ELM), and the bottom of the IS+OS where the OS intersects with the RPE. Raters performed segmentation across ∼0.5 mm of the *x*-axis of each B-scan for a final segmented volume of 0.5×0.5 mm for each eye. Retinal thickness was defined as the distance between the top of the retina and the intersection of the bottom of the retina and RPE (Fig. 3J). IS+OS thickness was defined as the distance between the intersection of the ELM and IS and the intersection of the OS and RPE (Fig. 3K). Measurements of the segmented images were performed in MATLAB. Data were calculated by one-way ANOVA followed by Dunnett's multiple comparisons test using GraphPad Prism version 7.00 for Mac (GraphPad Software).

### Electron microscopy

Eyes from WT and *Pgc-1α^+/−^* mice under RD or HFD were fixed in phosphate-buffered saline (PBS)-buffered glutaraldehyde (2.5% at pH 7.4). Eyes were cut at the posterior margin of the limbus and the cornea with scissors, then fixed one more time in PBS-buffered glutaraldehyde (2.5% at pH 7.4) and PBS-buffered osmium tetroxide (0.5%). The posterior segment of the eyes was embedded in epoxy resin (*n*=5 in each group). Thin sections (90 nm) were collected on 200-µm mesh copper grids, dried for 24 h and double-stained with uranyl acetate and lead citrate. Sections were viewed and imaged at 80 kV with a Hitachi HT-7700 transmission electron microscope.

### Paraffin histology

Eyes from WT and *Pgc-1α^+/−^* mice under RD or HFD were fixed in PBS-buffered paraformaldehyde (4% at PH 7.4) and embedded in paraffin. Thin sections of 6 µM were collected on slides and processed for Masson's Trichrome staining according to the established protocol (Cohen, 1976). This method is used for the detection of collagen fibers on formalin-fixed, paraffin-embedded sections: collagen fibers stain in blue, the nuclei in purple or black.

### Immunofluorescence staining

Eyes from WT and *Pgc-1α^+/−^* mice under RD or HFD were enucleated and were immediately submerged in optimal cutting temperature (OCT; Lab-Tek) and frozen in dry ice/ethanol for immunofluorescence staining. Frozen sections (6 μm) were rehydrated with 1×PBS, fixed with 4% PBS-buffered paraformaldehyde and permeabilized with PBS+0.1% Triton X-100. After 45 min blocking with BlockAid (Thermo Fisher Scientific), the sections were incubated with primary antibody overnight at 4°C. Sections were washed with PBS+0.5% BSA, then incubated with secondary antibody for 60 min at room temperature while protected from light. Slides were incubated with 0.05% Sudan Black (Sigma-Aldrich) in 70% ethanol for 35 min to decrease autofluorescence. Slides were mounted with Prolong Gold Antifade Mountant (Thermo Fisher Scientific) before imaging. Sections were viewed and imaged with the EVOS FL imaging system (Thermo Fisher Scientific). Primary and secondary antibodies were used according to the manufacturer's instructions and were validated before use in the experiments (Table S1).

### qPCR analyses

Mice were euthanized and eyes enucleated and immediately placed on gauze soaked with PBS. Eyes were cut at the posterior margin of the limbus and the cornea with scissors. The cornea, iris and lens were removed and discarded. The optic nerve was cut and the retina was carefully removed and preserved. The RPE/choroid/sclera was then transferred into a small Petri dish and was flash-frozen with liquid nitrogen. Using a 200 µl pipette and pipette tip, PBS was flushed into the eyecup to detach RPE. This step was repeated 20 times reusing the same PBS according to the established protocol (Xin-Zhao Wang et al., 2012). The isolate RPE and retina were transferred to a 2 ml centrifuge tube and processed for RNA or DNA extraction.

Total RNA was extracted with the RNeasy kit (Invitrogen), treated with RNase-free DNase I (Qiagen), and reverse transcribed with oligo-dT using the SuperScript III cDNA synthesis kit (Invitrogen). qPCR was performed with the Power SYBR™ Green Master Mix (Applied Biosystems). Specific primers (Table S2) for each gene were designed with the PrimerQuest software (Integrated DNA Technologies), and the cDNA sequences of each gene (GenBank) were used to produce 100-250 bp PCR amplicons that span one or more exon/intron boundaries. To validate the purity of the RPE/retina and efficient removal of the choroid, qPCR was performed with RPE65, rhodopsin and vascular endothelial cadherin (VE-cadherin). We observed expression of both Rpe65 and rhodopsin, but did not obtain detectable levels of VE-cadherin (Fig. S1).

### mtDNA copy number

The RPE/retina was isolated and processed for DNA extraction using DNA isolation kit (Thermo Fisher Scientific). The *Nd1* gene of mtDNA and the *H19* gene of nuclear DNA (nDNA) were amplified by qPCR (CFX Connect Real-Time PCR detection system); 2.5 ng template, 5 μl PowerUp SYBR Green (Thermo Fisher Scientific) and 300 nM primers in a total volume of 10 μl were used per reaction. The reaction was initiated at 50°C for 2 min, followed by 95°C for 2 min, then 40 cycles at 95°C for 1 s, 60°C for 30 s. All reactions were run in triplicate. Cycle threshold values were analyzed to determine the relative mtDNA to nDNA ratio for each sample.

### Mitochondrial complex I activity measurement

The RPE/retina was isolated and protein extracted in 100 μl T-PER buffer (Pierce Biotechnology). Tissues were homogenized with a glass tissue grind tube (Kontes) on ice. The supernatant was collected after centrifugation at 13,500 ***g*** for 10 min. The protein concentration was detected by BCA assay (Thermo Fisher Scientific) and 110 μg protein from each sample was used for the experiment. The activity of complex I was measured using a Complex I Enzyme Activity Kit (Abcam) according to the manufacturer's protocol. Absorbance at 450 nm was measured in a transparent flat-well plate at 30 s intervals for 30 min. The activity of complex I was presented as rate/slope (mOD/min) per 110 μg of protein.

### ROS measurement

The RPE/retina was isolated and proteins extracted using RIPA buffer; the protein concentration was measured using the BCA assay (Thermo Fisher Scientific). The ROS levels were measured with the In Vitro ROS/RNS Assay Kit (Cell Biolabs) according to the manufacturer's protocol. A 10 μg aliquot of protein from each sample, together with the standard curve made by a series of H_2_O_2_ dilutions, were incubated with 2′,7′-dichlorodihydrofluorescein (DCFH) for 30 min. Fluorescence was read at 480 nm excitation/530 nm emission with a plate reader (EnSpire multimode, PerkinElmer). ROS levels were calculated using the H_2_O_2_ standard curve, according to the manufacturer's protocol.

### Western blot analysis

Mice were euthanized and eyes enucleated and immediately placed on gauze soaked with PBS. Eyes were cut at the posterior margin of the limbus and the cornea with scissors. The cornea, iris and lens were removed and discarded. The optic nerve was cut and the retina was carefully removed and preserved. The RPE/choroid/sclera was cut in four slits with scissors to flatten the tissue and RPE cells were isolated using an established protocol (Wei et al., 2016). The RPE/retina was used for protein extraction followed by western blot analysis. To validate the purity of the RPE/retina tissue and efficient removal of the choroid, western blot analyses with RPE65 (a generous gift from Dr Michael Redmond, NEI/NIH), rhodopsin (Thermo Fisher Scientific) and VE-cadherin (R&D Systems) were performed (Fig. S1C,D). Protein samples were extracted in RIPA buffer (1% NP-40, 0.5% sodium deoxycholate, 0.1% SDS, 150 mM NaCl, 50 mM Tris-HCl in ddH_2_O) containing freshly added Protease and Phosphatase Inhibitor Cocktail Tablets (Invitrogen). Protein sample concentrations were measured by Pierce™ BCA Protein Assay Kit (Thermo Fisher Scientific) and analyzed using the NuPAGE electrophoresis and XCell Western Blot System (Invitrogen). Primary and secondary antibodies were used according to the manufacturer's instructions and were validated before use in the experiments (Table S1). Immunoreactive bands were visualized by the Clarity chemiluminescent substrate (Bio-Rad) followed by exposure to X-ray film. Densitometry was performed using ImageJ software.

### Statistical analyses

Quantitative immunoblot and gene expression assays were performed for each group of animals at least three times. Data presented as mean±s.e.m. of three independent experiments. The number of animals used in each experiment is noted as *n* under each group and was determined based on the feasibility criteria to provide necessary power for statistical analyses. Each sample is represented by three to six replicates per experiment for gene expression. One-way ANOVA followed by Dunnett's multiple comparisons test was performed using GraphPad Prism version 7.00 for Mac (GraphPad Software).

## Supplementary Material

Supplementary information
