# Supplementary material for: Pgc-1α repression and high-fat diet induce age-related macular degeneration-like phenotypes in mice
Source: Dis Model Mech. 2018 Aug 16;11(9):dmm032698. doi: 10.1242/dmm.032698 (PMC6176989; doi:10.1242/dmm.032698)
Supplement: Supplementary information [file dmm-11-032698-s1.pdf]

Supplementary Figure 1.

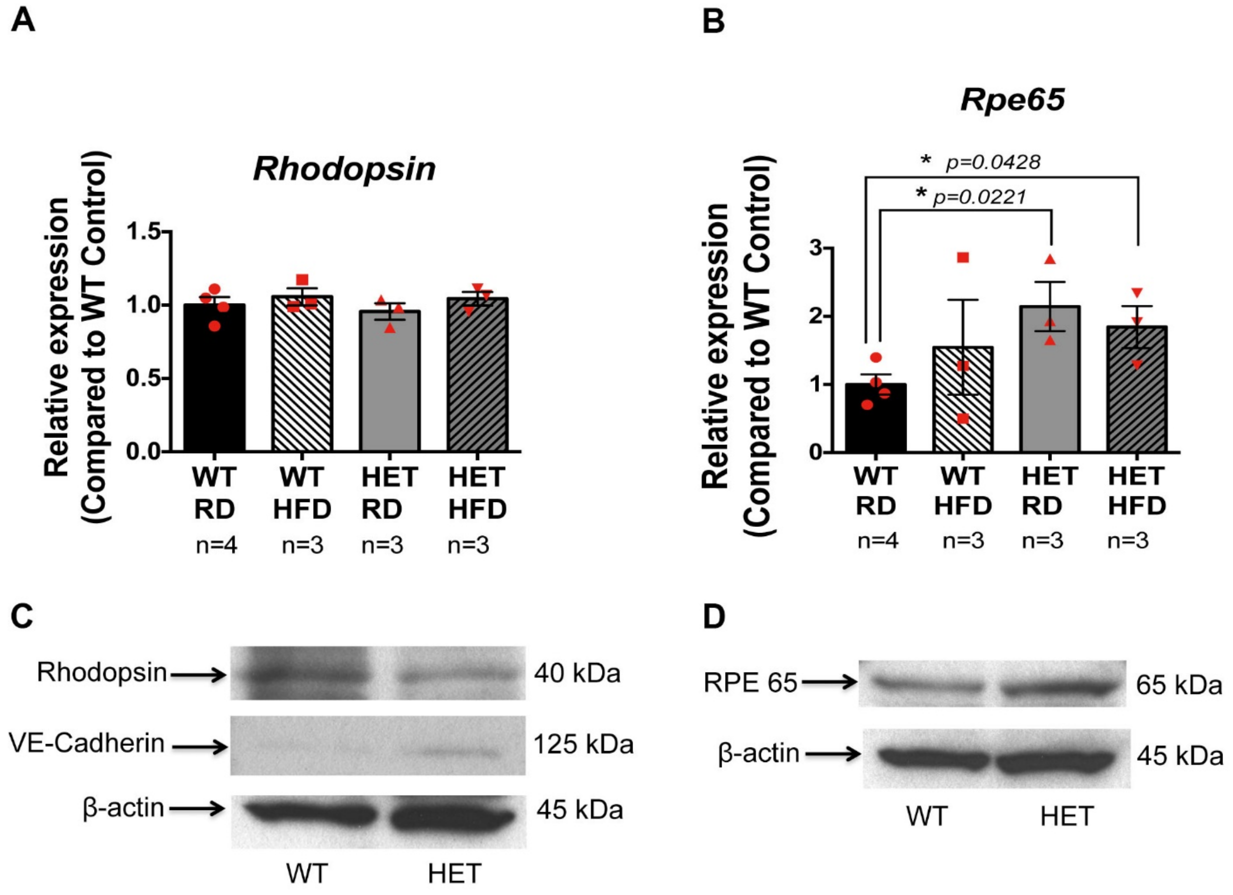

Supplementary Figure 1.

(**A**) Rhodopsin gene expression in RPE/retina of WT and *PGC-1α* $^{+/-}$ (HET) under RD and HFD.

(**B**) Rpe65 gene expression in RPE/retina of WT and *PGC-1α* $^{+/-}$ (HET) under RD and HFD showing increased *Rpe65* expression in the RPE/retina of *PGC-1α* $^{+/-}$ mice fed RD or HFD as compared to WT mice fed RD, suggesting that *Pgc-1α* might regulate *Rpe65* expression, or that increased levels of *Rpe65* expression might be a compensatory response to oxidative stress and photoreceptor degeneration. (*n* represents the number of mice in each group).

(**C**) Representative western blot image of rhodopsin, RPE65 and VE-cadherin showing rhodopsin and RPE65 protein expression and absence or minimal VE-cadherin expression in RPE/retina extract of WT and *PGC-1α* $^{+/-}$ (HET) mice.

Table S1. Antibodies used in this study.

Name of Antibody	Concentration	Reference#
LC3B Rabbit Ab	1:1,000	Cell signaling, 2775
SQSTM1/P62 Rabbit Ab	1:1,000	Cell signaling, 5114
β-Actin (D6A8) Rabbit mAb	1:10,000	Cell signaling, 8457
Rabbit IgG HRP-Linked Ab	1:3,000	Cell signaling, 7074
Goat IgG HRP-conjugated Ab	1:1,000	R&D System, HAF017
Mouse IgG, HRP-linked Ab	1:3,000	Cell signaling, 7076S
Bestrophin Rabbit Ab	1:500	Biorbyt, orb323221
Mouse VE-Cadherin Ab	0.2 µg/ul	R&D System, AF1002
Rhodopsin mouse mAb	1:1,000	Thermo Fisher, MA1-722
RPE65 Rabbit	1:500	A gift from Dr. M. Redmond laboratory, NEI/NIH
AGE, Carboxy-Methyl Lysine mouse mAb	1:100	Millipore, MABN1837
Goat anti-Mouse IgG (H+L) Cross-Adsorbed Secondary Ab	1:1000	Thermo Fisher, A-11001

Table S2. Primers used in this study.

Gene	Primer sequence	
Gapdh	Forward	AGACAGCCGCATCTTCTTGT
	Reverse	AATCTCCACTTTGCCACTGC
Tnfα	Forward	GTAGCCCACGTCGATGCAAA
	Reverse	ACAAGGTACAACCCATCGGC
Infγ	Forward	GGCAAAAGGATGGTGACATGA
	Reverse	TTTCGCCTTGCTGTTGCTGA
Pgc-1α	Forward	AGCCGTGACCACTGACAACGAG
	Reverse	GCTGCATGGTTCTGAGTGCTAAA
ApoE	Forward	GGTTCGAGCCAATAGTGGAA
	Reverse	ATGGATGTTGTTGCAGGACA
ApoJ	Forward	CAGCTGGCTAACCTCACACA
	Reverse	TGTGATGGGGTCAGAGTCAA
ApoB	Forward	GCCCATTGTGGACAAGTTGATC
	Reverse	CCAGGACTTGGAGGTCTTGGA
App	Forward	TGCAGCAGAACGGATATGAG
	Reverse	ACACCGATGGGTAGTGAAGC
Vegfa	Forward	AGCACAGCAGATGTGATTGC
	Reverse	TTTCTTGCGCTTTCGTTTTT
Rpe65	Forward	TGATGGTGTGGTTCTGAGTGTGGT
	Reverse	AAGAGGGCATTGGATTCCGTCTCA
Rhodopsin	Forward	TTCGTGGTCCACTTCACCATTCCT
	Reverse	TGATAACCATGCGGGTGACTTCCT
VE-Cadherin	Forward	CCGGCGCCAAAAGAGAGA
	Reverse	CTGGTTTTCCTTCAGCTGGAAGTGGT
Sod2	Forward	TCATGCATGCAAATCCTTGT
	Reverse	CCAGACCCAACAAGCTCTTC
Nd1	Forward	CTCTTATCCACGCTTCCGTTACG
	Reverse	GATGGTGGTACTCCCGCTGTA
H19	Forward	GTACCCACCTGTCGTCC
	Reverse	GTCCACGAGACCAATGACTG

Disease Models & Mechanisms • Supplementary information
